# Evaluation of a Bladder Cancer Cluster in a Population of Criminal Investigators with the Bureau of Alcohol, Tobacco, Firearms and Explosives—Part 2: The Association of Cancer Risk and Fire Scene Investigation

**DOI:** 10.1155/2013/986023

**Published:** 2013-04-13

**Authors:** Susan R. Davis, Xuguang Tao, Edward J. Bernacki, Amy S. Alfriend, Mark E. Delowery

**Affiliations:** ^1^Federal Occupational Health, United States Department of Health and Human Services, 4550 Montgomery Avenue, Suite 950, Bethesda, MD 20814, USA; ^2^Division of Occupational and Environmental Medicine, Department of Medicine, Johns Hopkins University School of Medicine, 600 N. Wolfe Street, Baltimore, MD 21205, USA

## Abstract

This study evaluated the association of bladder cancer risk and fire scene investigation within a cohort of white male criminal investigators with the United States Bureau of Alcohol, Tobacco, Firearms and Explosives that was found to be at increased risk for bladder cancer. Medical surveillance data were used in a nested case-control study to determine odds ratios (ORs) estimating the relative risk of the cancer associated with post-fire investigation. The study comprised seven bladder cancer cases and 1525 controls. Six of the cases reported holding assignments associated with post-fire investigation. The OR for bladder cancer was 19.01 (95% confidence interval = 1.94–186.39) for those holding any one or more of these assignments for one to four years versus zero years and 12.56 (1.14–138.58) for those holding any one or more of these assignments for five or more years versus zero years. The risk for bladder cancer is significantly elevated for those holding post-fire investigation assignments compared to those not holding these assignments.

## 1. Introduction

As presented in Part 1 of this epidemiologic study, a bladder cancer cluster occurred within a cohort of white male criminal investigators working with the Bureau of Alcohol, Tobacco, Firearms and Explosives (ATF), United States Department of Justice, between 1994 and 2005 [1]. The cluster was identified through the self-reporting of employees participating in a medical surveillance program which was initially set up to monitor the health of employees dedicated to the investigation of fires and explosions. There were seven self-reported cases in the bladder cancer cluster. Six of the seven were pathology report verified as urinary bladder cancer, five as low grade papillary transitional cell carcinoma, and one as transitional cell carcinoma in situ. In Part 1, analysis of bladder cancer incidence in the study population determined that white male criminal investigators, without regard to work history, were at statistically significant increased risk for bladder cancer [1]. Because six of the seven cases reported holding special assignments associated with post-fire/post-blast scene investigation while employed with ATF, the bladder cancer cluster appeared to be associated with the work on such scenes.

Most scenes investigated by ATF are post-fire rather than post-blast and involve municipal structures. Although ATF employees who investigate post-fire scenes typically wait until the fire is out to enter the scene to search for origin and cause, the work still puts them at risk for exposure to a mix of hazardous chemicals and products of incomplete combustion which potentially includes known and suspect bladder carcinogens such as aromatic amines and polycyclic aromatic hydrocarbons (PAHs) [2–20]. Guidotti and Clough [21] expressed particular concern for exposures during the clean-up phase related to the smoldering of synthetic materials and the release of trapped gasses from porous materials, and this concern applies to fire investigation activities. Several authors offer detailed reviews of the general toxicologic aspects of fire smoke and summaries of findings of fire scene exposure monitoring projects [21–23]. Most recently, the International Agency for Research on Cancer published a monograph on firefighting which focuses on the results of studies measuring exposures to carcinogens found in smoke at fires [24]. Studies attempting to characterize exposures during firefighting activities have in general not monitored for aromatic amines and relatively few have monitored for PAHs [22–27]. As critiqued by Golka and Weistenhöfer [23], the studies on characterization of smoke at fire scenes do not support the premise that firefighters are at increased risk for bladder cancer.

 Independently, ATF sponsored two efforts to characterize exposures occurring during fire scene investigation by ATF employees, (1) a health hazard evaluation performed by NIOSH in 1997 [28] and (2) a comprehensive industrial hygiene study carried out by a certified industrial hygienist between 2005 and 2009 (F. Fitzpatrick, CIH, unpublished data provided in report to ATF entitled “Exposures to Chemicals and Other Hazardous Substances During ATF Post-Fire/Blast Scene Investigations,” May 2010). Both studies monitored for exposure to PAHs, and the more recent study also monitored for aromatic amines. The first study monitored five different fire scenes, while the second study monitored 13 different fire or blast scenes. Low levels of PAHs were obtained at fires scenes in both studies, and no detectable levels of aromatic amines were obtained in the second study. In both studies all detectable levels of PAHs were well below established occupational exposure limits, and none of the detectable PAHs in Fitzpatrick's study were classified as A1 or A2 carcinogens by the American Conference of Governmental Industrial Hygienists. 

 As no two fire scenes are alike and specific exposure risks vary from one scene to another, these industrial hygiene findings may not typify investigator exposures at all present-day fire scenes and may not characterize exposures occurring at fire scenes 10 to 20 years ago. Investigator variables such as time spent on scene, use of personal protective equipment, and adherence to cleanup procedures will also influence exposure risks. Potential for internal exposure to PAHs during firefighting activities has been demonstrated in a study on firefighters exposed to burning diesel fuel during training exercises [29]. This study found slight but statistically significant increases in PAH urine levels over several days of fire suppression training, reductions in levels with use of respirators, and higher levels among smokers [29], and may have application to the job of post-fire investigation. Since regular and recurring work as a fire investigator poses increased risk for exposure to variable and mixed products of combustion, such work might also be associated with increased risk for bladder cancer. 

No epidemiologic study on bladder cancer risk in fire investigators has been previously reported. Since firefighters and fire investigators share a potential for similar exposures, and firefighters may participate in fire investigation, a review of the epidemiologic studies on bladder cancer in firefighters is appropriate. Since the mid-1980s numerous epidemiologic studies [30–44] and review articles [6, 23, 24, 45] have explored the relationship between firefighting and bladder cancer risk. At least 10 studies have looked at cancer mortality [30–32, 34–36, 38, 40, 41, 44], and six studies have looked at cancer incidence [32, 33, 37, 39, 42, 43]. In addition, other epidemiologic studies, evaluating the association of bladder cancer incidence and occupations in general, have also addressed the cancer risk in firefighters [46–50]. There have been inconsistencies in results among these studies, and in those studies showing an increased risk of bladder cancer incidence or mortality among firefighters, few achieved statistical significance. These included one mortality cohort study with a standardized mortality ratio (SMR) of 2.86 (95%CI 1.30–5.40) [44], one incidence case-control study with an odds ratio (OR) of 1.59 (95%CI 1.02–2.50) [43], and one incidence cohort study with a standardized incidence ratio (SIR) of 1.29 (95%CI 1.10–1.62) [42]. Three recently published meta-analyses [51–53] and one unpublished meta-analysis (X. Tao, MD, PhD, unpublished data provided in report to ATF entitled “Evaluation of a Bladder Cancer Cluster among a Cohort of Criminal Investigators with the Bureau of Alcohol, Tobacco, Firearms and Explosives,” November 2007) of the combined findings of epidemiologic studies addressing firefighters and bladder cancer risk found a 1.17-fold to 1.39-fold increase in cancer incidence risk and a 1.14-fold to 1.29-fold increase in cancer mortality risk, but these increases were of no or only marginal statistical significance. In the most recently published meta-analysis of bladder cancer incidence in firefighters, which included nine studies, the summary relative risk (SRR) was 1.17 (95%CI 0.92–1.49) [52].

Part 2 of this study uses data from the ATF medical surveillance program to evaluate the association between post-fire/post-blast investigation and bladder cancer risk within the study's white male cohort through an internal nested case-control analysis which controlled for both age and tobacco use history.

## 2. Methods

### 2.1. Study Time Frame and Study Subjects

Part 1 [1] previously detailed the ATF medical surveillance program, which was established on a voluntary basis in 1995 and became mandatory in 2002, and the full roster cohort of 3768 employees, predominantly criminal investigators, used in the bladder cancer incidence study. As 2003 was the first year of the program in which participating ATF employees completed detailed work history questionnaires, this analysis focused on program participants between 2003 and 2007.

 All seven bladder cancer cases identified in the ATF cohort study of Part 1 [1] were self-reported by employees participating in the medical surveillance program. Verification of six of the seven cancers by pathology report was addressed in Part 1 [1]. As all reported bladder cancers occurred in white males, this study was limited to white males. The cases for this analysis, by definition, included all individuals with self-reported bladder cancers who were employed by ATF at the time of diagnosis and who had at least one medical surveillance exam during the period 2003–2007 with complete data on all study parameters. 

The selected control group, by definition, included all white males in the ATF cohort of Part 1 who had at least one medical surveillance exam during the period 2003–2007 with complete data on all study parameters and no self-reported history of any type of cancer. Thus, study controls were representative of all noncases in the population. 

### 2.2. Age Parameter

Since risk for bladder cancer increases with age, this study controlled for age. Dates of birth for cases and controls were self-reported through the medical surveillance program and then verified through cross-referencing with dates of birth obtained from annual ATF staffing rosters. Year of diagnosis of bladder cancer for cases was self-reported through the medical surveillance program and then verified through cross-referencing with a pathology report, as available. For study purposes, case age was the age the case turned in the year of diagnosis and control age was the age the control turned in the year of the most recent complete examination in the database. 

### 2.3. Tobacco Use Parameters

As cigarette smoking is a significant risk factor for bladder cancer, this study controlled for tobacco use history. Tobacco use history for cases and controls was self-reported through the medical surveillance program and included the following: (1) yes or no if tobacco products were ever used, (2) whether use was current or past, (3) if past, the year quit, (4) type of product used (cigarettes, pipe/cigar, snuff/chew), (5) number of years used, and (6) amount per day. For study purposes, tobacco use data for the cases reflected tobacco use status in the year of diagnosis and tobacco use data for the controls reflected tobacco use status in the year of the most recent complete examination in the database. For cases diagnosed prior to 2003, historical medical surveillance data was accessible to verify tobacco use status in the year of diagnosis. Tobacco use status of cases after year of diagnosis was excluded from study as it was not relevant.

### 2.4. Work History Parameters

The selected work history parameters for this study included (1) number of years reported working on team assignments and with special designations relevant to post-fire/post-blast investigation (special assignment years), (2) number of years reported working post-fire/post-blast scenes (fire scene exposure years), and (3) number of days reported working post-fire/post-blast scenes (fire scene exposure days). As ATF investigations are predominately post-fire, these work history parameters serve as surrogate measures or variables of exposure to products of combustion associated with fire scenes. 

These parameters applied only to work with ATF and did not include work with prior employers. The team assignment and special designation categories included (1) National Response Team (NRT), (2) Division Response Team (DRT), (3) Arson Task Force, (4) Certified Fire Investigator (CFI), and (5) Certified Explosives Specialist (CES). The terms NRT, CFI, and Arson Task Force were clarified in Part 1 of this study [1]. The DRT is similar in concept to the NRT but responds to requests for assistance on a regional level rather than a national level. CESs are criminal investigators who have gone through a special training to receive certification as explosives experts. 

Work history information was collected through the medical surveillance program in the same way for both cases and controls. For cases, work history data reflected the reported number of years worked or number of days worked up to the year of diagnosis. A projection was used to calculate the number of days worked on fire and explosives scenes up to the year of diagnosis, if this information was first reported after the diagnosis year. This projection was based on the assumption that the days worked were evenly distributed over the years worked on fire scenes. For controls, work history data reflected the reported number of years worked or number of days worked up to the year of the most recent complete examination available in the database.

### 2.5. Study Design

Evaluation of the association between post-fire/post-blast scene investigation and bladder cancer incorporated a nested case-control study design to compare the work histories of the bladder cancer cases and the work histories of the controls with no history of any cancer. As stated earlier, since all identified bladder cancers in the study population occurred in white males, the nested case-control study focused only on white males. See Section 2.1 for previously provided definitions of the cases and the controls used in this analysis.

Odds ratios (ORs), based on logistic regression models, were computed to estimate the relative risk of bladder cancer associated with each fire scene exposure study parameter, while controlling for the confounding factors tobacco use and age. Analyses of the special designation and team assignment categories included both years worked in each category and years worked in any one or more of the five categories combined. 

Controls were not matched with cases in terms of the exposure parameters under study in order to avoid overmatching bias. To control for age, cases and controls were grouped into 10-year age increments: less than 30 years, 30–39 years, 40–49 years, 50–59 years, and 60 or more years. To control for tobacco use, cases and controls were grouped into the following categories: non-user, user less than 10 years, and user 10 or more years. The small sample size limited more sophisticated covariate matched analysis involving age or further stratified analysis of tobacco use history.

## 3. Results

### 3.1. Study Subjects

During the period 2003–2007, 2549 members (68%) of the full roster cohort of 3768 individuals (previously detailed in Part 1 [1]) completed at least one examination and one work history questionnaire. These 2549 individuals represented 87% of the 2928 cohort members who were employed by ATF for one or more years during 2003–2007. 

Table 1 shows the distribution of individuals by race and sex for these 2549 individuals, as well as the distribution of self-reported bladder cancers and all other cancers, by race and sex. All seven of the self-reported white male bladder cancers of the cohort study met the definition as a case for this study and had complete study parameter data. Of the 1771 white males in the population of Table 1, 1525 individuals met the definition as a white male control and had complete study parameter data. Excluded from the control group were 79 white males with a reported history of some other cancer and 160 white males with incomplete study parameter data. Thus, the study population for the internal comparison analysis comprised seven reported bladder cancer cases and 1525 controls with no history of cancer. All cases and 97% of controls were criminal investigators. The remaining controls were primarily explosives enforcement specialists, forensic chemists, and fire protection engineers, typical members of the NRT.

### 3.2. Characterization of Cases and Controls by Age and Tobacco Use History

Table 2 shows the distributions of cases and controls among the age increments and tobacco use parameters selected for analysis. The percent distributions of cases and controls in each 10-year age category were similar. About the same percentage of cases and controls was also tobacco nonusers, but the percentage of cases with less than 10 years of tobacco use was about half the percentage of controls with less than 10 years of tobacco use, and the percentage of cases with 10 or more years of tobacco use was about twice the percentage of controls with 10 or more years of tobacco use. By controlling for any form of tobacco use rather than cigarette use alone, greater weight was given to the cancer risk attributed to tobacco use, and lesser weight was given to the cancer risk associated with fire investigation work. 

### 3.3. Characterization of Cases and Controls by Distribution of Exposure Variables

Among the seven cases, six reported work histories associated with investigation of fire scenes while employed with ATF, as mentioned in the introduction. These six cases also comprised the six cancer cases verified by pathology report. At the time of diagnosis, three cases were both CFIs and members of the NRT, one was a CFI but not a NRT member, one was a member of the NRT but not a CFI, and one was a member of the Division Response Team (DRT). Three cases were also members of the Arson Task Force and two of the CFIs with membership on the NRT were also CESs. None of the seven reported work histories associated with fire scene investigation prior to employment with ATF.

Table 3 shows the distributions of cases and controls for each analyzed work parameter. For fire scene exposure days, a considerably lower percentage of cases reported 1 to 199 days of exposure compared to their control counterparts, and a considerably higher percentage of cases reported 200 or more days of exposure compared to their control counterparts. For fire scene exposure years, the distributions of cases and controls among the parameter's incremental categories were fairly similar. For the study parameters of years spent on special assignment, when applicable, the percentages of cases with one to four years and with five or more years on special assignment exceeded the respective percentages of controls for any one or more of the special assignments combined and for each individual assignment.

### 3.4. Logistic Regressions of Exposure Variables

Table 4 presents the odds ratios (ORs) for each exposure variable, while controlling for tobacco use and age. Analysis of fire scene exposure days did not show a statistically significant increase in bladder cancer risk with either one to 199 days or 200 or more days on fire scenes compared to zero days on fire scenes and actually suggested a protective effect with one to 199 days of exposure compared to zero days (OR 0.05 (95%CI 0.00–0.82)). Likewise, analysis of fire scene exposure years did not show a statistically significant increase in bladder cancer risk with either one to nine years or 10 or more years on fire scenes compared to zero years on fire scenes. 

Analyses of years spent on any one or more of the special assignments combined and on each individual special assignment did, however, identify significant associations between special assignment work and increased risk for bladder cancer. For participation on any one or more of the special assignments, the OR was 19.01 (95%CI 1.94–186.39) for one to four years on any special assignment compared to zero years and 12.56 (95%CI 1.14–138.58) for five or more years compared to zero years. For individual team assignments, NRT work with both one to four years and five or more years, DRT work with five or more years, and arson task force work with one to four years exposure were all associated with statistically significant increase in bladder cancer risk. The CFI designation was associated with the highest ORs for both one to four years and five or more years compared to zero years. The CES designation was the only individual special assignment which was not associated with increased risk of bladder cancer. 

## 4. Discussion

As previously detailed in Part 1 of this epidemiologic study, a bladder cancer cluster occurred within a cohort of white male criminal investigators working with ATF between 1994 and 2005 [1]. The cluster consisted of seven self-reported cases, of which six were verified by pathology report. All six of the verified cases also had work histories of participation in fire scene investigations. This observation raised concern that fire scene investigation might be linked to increased risk for bladder cancer in this population. The nested case-control study of Part 2 shows a statistically significant association between work on special assignments involving post-fire scene investigation and increased risk for bladder cancer and complements the findings of Part 1, which show a significant increase in bladder cancer incidence in the criminal investigator employee population. No previous study reported in the literature has addressed the association of bladder cancer risk and fire investigation.

Specifically, the nested case-control analysis showed participation on any one or more of the special assignments combined to be associated with a greater than 12-fold increase in bladder cancer risk for both one to four years and five or more years of exposure, when compared to zero years of exposure, as detailed in Section 3.4. With individual special assignments, greater than ninefold increases in cancer risk were seen for NRT members and for CFIs with both one to four years and five or more years exposure, for DRT members with five or more years exposure, and for Arson Task Force members with one to four years exposure, when compared to zero years exposure. Only the CES assignment, the one typically least involved with fire scene investigation, was not associated with statistically significant increase in risk of bladder cancer. The greatest increase in cancer risk was associated with the CFI special assignment, which was held by four of the seven bladder cancer cases, with ORs of 43.84 (95%CI 6.70–287.02) for one to four years and 22.76 (95%CI 2.52–205.91) for five or more years. This finding is likely explained by the focused and intense role CFIs play in fire investigation. Also noteworthy, since individuals serving in any one of these special assignments frequently participate in one or more of the other special assignments, the individual assignment categories are not independent of one another. Consequently, the ORs for participation on any one or more of the special assignments combined may best characterize general estimations of cancer risk associated with fire scene investigation. Another interesting point is the finding that the ORs for one to four years of exposure were greater than the ORs for five or more years of exposure for the NRT, CFI, and any one or more of the special assignments combined analyses. This observation suggests a very short latency period for some of the cases and may pertain to the requirement for CFI candidates to participate in 100 fire scene investigations during the CFI two-year certification process. It should also be mentioned that, aside from the statistical significance of the ORs found with the special assignment analyses, the observed wide confidence intervals limit any perceived importance of differences in the magnitude of the ORs between assignment categories or between one and four years of exposure and five or more years of exposure. 

As addressed in the introduction, known and suspect bladder carcinogens are potentially present in postcombustion products present at fire scenes, and the threat for exposure to postcombustion products from smoldering and off-gassing materials exists during investigation of those scenes. Primary routes of exposure to these products include inhalation and skin absorption. Exposure risk is dependent on a variety of factors including scene parameters (e.g., presence of smoldering hot pockets, amount of ventilation) and work practices (e.g., use of personal protective equipment, eating and drinking on site, maintaining adequate hydration, containment and decontamination of soiled clothing and equipment, timeliness of personal cleanup upon leaving the scene). While the use of self-contained breathing apparatus (SCBA) and firefighter turnout gear is an established practice during fire suppression, the use of respirators and other personal protective equipment during post-fire overhaul and investigation activities is less routine and is generally based on a judgment call made at the scene. As such, exposure to hazardous chemicals may in fact be greater during post-fire overhaul and investigation than during fire suppression. Some fire investigators with ATF have in the past described clearing their nose of “black mucus” for several days following a three to five day post-fire investigation or experiencing their vehicles reeking of smoke from soiled clothing and equipment. This anecdotal information appears to support the findings of this nested case-control study by illustrating that fire investigators could potentially be exposed to bladder carcinogens through both inhalation and skin absorption. Even though the air-monitoring industrial hygiene studies reviewed in the introduction did not find bladder carcinogen concentrations of concern, the studied fire scenes may not represent all fire scenes and may not adequately define investigator risk for exposure to bladder carcinogens, especially historical risk occurring during the years prior to bladder cancer diagnosis. With each fire scene being unique, investigator use of respirators and other personal protective equipment, adherence to housekeeping measures related to personal hygiene and cleanliness, and decontamination of clothing and gear can be expected to moderate potential internal exposure to combustion products and any associated cancer risk. After concern was first raised that fire investigation appeared to be associated with increased risk for bladder cancer, ATF elected to take precautionary actions to raise awareness among investigators, formalize a respiratory protection program, and improve work practices associated with fire investigation to reduce potential for exposure to hazardous chemicals while at and upon leaving fire scenes. It has now been over six years since the most recently reported case of bladder cancer.

This is the first known epidemiologic study to evaluate the association of bladder cancer risk and fire scene investigation. The odds ratios generated in this study are relatively high when contrasted with findings of individual epidemiologic studies of bladder cancer risk in other occupations and industries. In these other studies, statistically significant increases in bladder cancer risk are typically found in the 1.1-fold to fivefold range but also occur in the sixfold to tenfold range, as addressed in the discussion section to Part 1 [1]. For example, one study of occupational factors and bladder cancer incidence in Canada showed statistically significant ORs for jobs in the chemicals industry (2.37), with tars or asphalt exposure (3.11), in dye manufacturing (3.62), and in the dyeing of cloth (4.63) [54]. Another case-referent study on occupational risk factors for bladder cancer in southern Israel found statistically significant ORs of 4.67 and 6.25 for occupational exposures to dusts and to multiple chemicals, respectively [55]. In one study on chimney sweeps, the standardized morbidity ratio for bladder cancer was elevated and statistically significant at 2.36 [8]. In the recent meta-analysis by Reulen et al. [52], however, statistically significant elevations in summary relative risks for bladder cancer, which were found for several occupations, were relatively low, in the 1.1-fold to 1.3-fold range, as explained in Part 1 [1]. The elevated ORs of the current study even exceed the twofold to sixfold increase in risk for bladder cancer typically found in cigarette smokers compared to nonsmokers [56–59]. Additional epidemiologic studies are warranted to corroborate the findings of this study and in particular the magnitude of the elevated risk found with fire scene investigation. 

In the interim, the findings of both the incidence study and the nested case-control study support ATF's preliminary initiatives to educate the employee population regarding the potential cancer risks associated with post-fire investigation, monitor the health of the employee population through the medical surveillance program, perform bladder cancer screening as part of the medical surveillance program, provide appropriate personal protective equipment to those investigating fire scenes, and establish guidance for appropriate cleanup and decontamination measures following fire scene work. Continued monitoring of current employees through the medical surveillance program for another five to 10 years is warranted to track the future pattern of bladder cancer occurrence in the population. In addition, with cancers being typically associated with latency periods, inclusion of retirees in some form of health-monitoring program should be a consideration. 

One strength of this nested case-control study is that work history data were available on 87% of the 2928 members of the full roster cohort who were employed with ATF for one or more years during the period 2003–2007, when the work history questionnaire was part of the medical surveillance program. With this level of participation in the program, any significant bias in study outcome from nonparticipants is unlikely. 

Another strength of the study is that the ATF employee population under study was fairly stable during the time frame of both the cancer incidence study of Part 1 [1] and the case-control study of Part 2. Although the seven individuals with reported bladder cancer were diagnosed over a 12-year period, 1994–2005, while employed with ATF, all were still employed with ATF during the period 2003–2007 and completed at least one work history questionnaire. In addition, of all white males in the full roster cohort of the incidence study [1], 65% were still employed with ATF during 2003–2007 and completed work history questionnaires. The actual control group for the nested case-control study comprised 56% of all white males in the full roster cohort of the incidence study.

The small number of cases is a limitation of this study and can be expected to contribute to statistical instability and wide confidence intervals of the ORs, but the large size of the control group counters the effect of the small case size and restores some statistical stability to the ORs or the confidence intervals would be even wider.

Another limitation of this nested case-control study concerns employee self-reporting of bladder cancer diagnoses. As presented in a prospective cohort study by Bergmann et al. [60] on the accuracy of self-reported cancer diagnoses when compared to state cancer registries, the sensitivity of self-reporting bladder cancer was 0.67 and the positive predictive value was 0.72. Although the sensitivity in the Bergmann study was only 0.67, the seven reported cases of the ATF cohort were sufficient in number to achieve statistically significant elevations in the SIRs computed in Part 1 [1] and in the ORs of this study associated with fire scene special assignments. Even though six of the seven identified cancer cases reported work on fire scene special assignments, any selective underreporting of bladder cancer among those with no fire scene special assignments would clearly affect the OR outcomes. From a positive predictive value perspective, six of the seven (86%) reported cases in this study were pathology report verified. Interestingly, the one unverified case was the same case with no reported work on fire scenes; even with inclusion of this unverified case, the ORs for the majority of fire scene special assignment analyses were significant. 

Employee self-reporting of all exposure parameters, with potential for recall bias, also presents a study limitation. In this study, several work history parameters were selected for evaluation as surrogate measures of exposure to products of combustion associated with fire scenes. The most reliably reported work parameter is conceivably the number of years spent working on special assignments associated with fire scene investigation, where assignment membership is formally established. This work parameter was the only one in the study found to be associated with statistically significant increased bladder cancer risk and appears to qualify as a surrogate measure of fire scene exposure. The reported number of years spent working on fire scenes might also be a reliably reported work parameter, but unlike the special assignment parameter, it may not have functioned as a good indicator of actual fire scene exposure, for there was not a significant association between fire scene years and increased cancer risk. The number of fire scene days is likely the most unreliably reported work parameter due to recall bias and employee retrospective estimation of days spent on fire scenes, especially for work predating the institution of the work history questionnaire in 2003. In addition, for the OR analysis of bladder cancer risk associated with reported fire scene days, as six of the seven cases were diagnosed prior to initiation of the work history form, the number of accrued days on fire scenes at the time of diagnosis was retrospectively calculated for these six cases through a systematic approach which applied the assumption that total accrued days were evenly distributed over the years worked on fire scenes with ATF. Inaccuracies in reported number of fire scene days and calculation assumptions could account for the lack of association between this work parameter and increased risk of bladder cancer and for the apparent protective effect of working 1–199 days on fire scenes versus zero days, as noted in Section 3.4, or this exposure variable may just not have been an appropriate surrogate of actual fire scene exposures. 

 To conclude, in this nested case-control study on the ATF medical surveillance population, white males with work histories of holding special assignments associated with post-fire/post-blast investigation had statistically significant elevated risk of bladder cancer compared to white males with no work histories of holding these special assignments. The CFI special assignment was associated with the greatest increased risk in bladder cancer. The other work parameters, days spent and years spent working fire scenes, were not associated with statistically significant increased risk for bladder cancer compared to no days spent and no years spent working fire scenes, respectively.

## Figures and Tables

**Table 1:** Distribution of all self-reported cancers by gender and race among employees with surveillance examinations and work histories.

Race	Male			Female			Total			
	Bladder cancer	Other cancers	Number of employees	Bladder cancer	Other cancers	Number of employees	Bladder cancer	Other cancers	Number of employees	Percent of full roster*
White	7	79	1771	0	13	253	7	92	2024	65.7%
Nonwhite	0	11	441	0	1	84	0	12	525	76.4%

Total	7	90	2212	0	14	337	7	104	2549	67.6%

*The full roster consisted of 3768 individuals of which 3081 were white and 687 were nonwhite.

**Table 2:** Distribution of age and tobacco use history among cases and controls.

	Cases (*N* = 7)		Controls (*N* = 1525)	
	*N*	%	*N*	%
Age				
<30 years	0	0%	85	5.5%
30–39 years	3	43%	625	41.0%
40–49 years	3	43%	640	42.0%
50–59 years	1	14%	169	11.1%
≥60 years	0	0%	6	0.4%
Tobacco use				
None	3	43%	715	46.9%
<10 years	1	14%	506	33.2%
≥10 years	3	43%	304	19.9%

**Table 3:** Distribution of fire scene work history parameters among cases and controls.

	Cases (*N* = 7)		Controls (*N* = 1525)	
	*N*	%	*N*	%
Fire scene exposure days				
0 days	1	14%	73	4.8%
1–199 days	1	14%	1270	83.3%
200 or more days	5	71%	182	11.9%
Fire scene exposure years				
0 years	1	14%	208	13.6%
1–9 years	3	43%	785	51.5%
10 or more years	3	43%	532	34.9%
Any special assignment* years				
0 years	1	14%	1034	67.8%
1–4 years	3	43%	179	11.7%
5 or more years	3	43%	312	20.5%
NRT years				
0 years	3	43%	1329	87.2%
1–4 years	2	28%	83	5.4%
5 or more years	2	28%	113	7.4%
DRT years				
0 years	4	57%	1434	94.0%
1–4 years	1	14%	39	2.6%
5 or more years	2	28%	52	3.4%
Arson task force years				
0 years	3	43%	1320	86.6%
1–4 years	4	57%	109	7.1%
5 or more years	0	0%	96	6.3%
CFI years				
0 years	3	43%	1440	94.4%
1–4 years	2	28%	20	1.3%
5 or more years	2	28%	65	4.3%
CES years				
0 years	5	71%	1297	85.1%
1–4 years	0	0%	54	3.5%
5 or more years	2	28%	174	11.4%

*“Any special assignment” is a special assignment summary parameter designating the holding of any one or more of the five individual special assignments combined.

**Table 4:** Odds ratios for bladder cancer risk associated with fire scene exposure parameters, while controlling for age and tobacco use.

Exposure parameter	Odds ratio (95% CI)	*P* value
Fire scene days		
1–199 days versus 0 days	0.05 (0.00–0.82)	0.04
200 or more days versus 0 days	4.50 (0.38–53.06)	0.23
Fire scene years		
1–9 years versus 0 years	0.78 (0.08–7.61)	0.83
10 or more years versus 0 years	1.22 (0.09–17.08)	0.88
Any special assignment* years		
1–4 years versus 0 years	19.01 (1.94–186.39)	0.01
5 or more years versus 0 years	12.56 (1.14–138.58)	0.04
NRT years		
1–4 years versus 0 years	12.74 (2.02–80.31)	0.01
5 or more years versus 0 years	9.87 (1.32–73.81)	0.03
DRT years		
1–4 years versus 0 years	8.21 (0.88–76.40)	0.06
5 or more years versus 0 years	12.71 (2.10–77.00)	0.01
Arson task force years		
1–4 years versus 0 years	16.88 (3.70–76.99)	0.0003
5 or more years versus 0 years	—	—
CFI years		
1–4 years versus 0 years	43.84 (6.70–287.02)	<0.0001
5 or more years versus 0 years	22.76 (2.52–205.91)	0.01
CES years		
1–4 years versus 0 years	3.15 (0.50–19.93)	0.22
5 or more years versus 0 years	0.47 (0.05–4.50)	0.51

*“Any special assignment” is a special assignment summary parameter designating the holding of any one or more of the five individual special assignments combined.
